# Supplementary material for: A Synthetic Consensus Chiroptera IFN-β Induces IFN-I Signaling Across Diverse Bat Taxa
Source: bioRxiv. 2026 Oct 29:2026.10.24.761547. Preprint. [Version 1] doi: 10.64898/2026.10.24.761547 (PMC13733770; doi:10.64898/2026.10.24.761547)
Supplement: Supplementary file 1 [file media-1.pdf]

Supplementary Materials for

A Synthetic Consensus Chiroptera IFN-β Induces IFN-I Signaling Across Diverse Bat Taxa

Julianne R. Bullock[1†], Brown Bulloch[1†], Anthony McBain[1], Tom Hill[2], Aaron Carmody[3], Yi He[4], Lucas De Sousa Paula[5], Paul Beare[2], Craig Martens[2], Arthur Wickenhagen[1], Sarah van Tol[1]*

This file includes:

Supplementary Table 1. Bat species used to design the consensus Chiroptera IFN-β sequence
Supplementary Table 7. List of genomes used in the OrthoFinder analysis
Supplementary Table 9. List of primers and probes for the target genes to evaluate host gene expression changes in *Artibeus jamaicensis* cells
Supplementary Figure 1. Generation of recombinant bat IFN-βs
Supplementary Figure 2. Determination of functionally equivalent doses to compare interferon stimulated gene repertoires
Supplementary Figure 3. Differentially induced genes share similar expression patterns across the IFN-I treatment groups
Supplementary Figure 4. Chiroptera IFN-β induces IFN-I signaling in cell lines from diverse bat taxa
Supplementary Figure 5. Chiroptera IFN-β induces an antiviral state in cell lines from diverse bat taxa
Supplementary Figure 6. Gating strategy to evaluate infection with VSV- or EBOV-GFP

Supplementary Tables Attached as Separate Excel File due to Size:

Supplementary Table 2. Table of differentially expressed genes identified following stimulation with the different IFN-I types
Supplementary Table 3. Gene ontology (GO) terms and descriptions of biological processes differentially regulated following AjKi2 cells stimulated with conspecific IFN-β
Supplementary Table 4. Gene ontology (GO) terms and descriptions of biological processes differentially regulated following AjKi2 cells stimulated with Phyllostomid consensus IFN-β
Supplementary Table 5. Gene ontology (GO) terms and descriptions of biological processes differentially regulated following AjKi2 cells stimulated with consensus Chiroptera IFN-β
Supplementary Table 6. Gene ontology (GO) terms and descriptions of biological processes differentially regulated following AjKi2 cells stimulated with Universal IFN-I
Supplementary Table 8. List of proteins used to generate a species-based tree

Supplementary Table 1. Bat species used to design the consensus Chiroptera IFN-β sequence

Sub-order	Family	Species	Genome Accession
Yangochiroptera	Miniopteridae	Miniopterus natalensis	GCA_001595765.1
		Miniopterus schreibersii	GCA_004026525.1
	Molossidae	Molossus molossus	GCA_014108415.1
		Tadarida brasiliensis	GCA_004025005.1
	Mormoopidae	Mormoops blainvillei	GCA_004026545.1
		Pteronotus mesoamericanus	GCA_021234165.1
		Pteronotus parnellii	GCA_000465405.1
	Noctilionidae	Noctilio leporinus	GCA_004026585.1
	Phyllostomidae	Anoura caudifer	GCA_004027475.1
		Artibeus jamaicensis	GCA_021234435.1
		Carollia perspicillata	GCA_004027735.1
		Desmodus rotundus	GCA_022682495.2
		Macrotus californicus	GCA_007922815.1
		Micronycteris hirsuta	GCA_004026765.1
		Phyllostomus discolor	GCA_004126475.3
		Phyllostomus hastatus	GCA_019186645.2
		Tonatia bakeri	GCA_004024845.1
	Vespertilionidae	Eptesicus fuscus	GCA_000308155.1
		Ia io	GCA_025583905.1
		Lasiurus borealis	GCA_004026805.1
		Lasiurus cinereus	GCA_011751095.1
		Murina feae	GCA_004026665.1
		Myotis brandtii	GCA_000412655.1
		Myotis davidii	GCA_000327345.1
		Myotis lucifugus	GCA_000147115.1
		Myotis myotis	GCA_014108235.1
		Nycticeius humaeralis	GCA_007922795.1
		Pipistrellus kuhlii	GCA_014108245.1
		Pipistrellus pipistrellus	GCA_903992545.1
		Pipistrellus pygmaeus	GCA_004026625.1
Yinpterochiroptera	Pteropodidae	Cynopterus brachyotis	GCA_009793145.1
		Eidolon helvum	GCA_000465285.1
		Eonycteris spelaea	GCA_003508835.1
		Macroglossus sobrinus	GCA_004027375.1
		Pteropus alecto	GCA_000325575.1
		Pteropus medius	GCA_902729225.1
		Pteropus pselaphon	GCA_014363405.1
		Pteropus vampyrus	GCA_000151845.2
		Rousettus aegyptiacus	GCA_014176215.2
		Rousettus leschenaultii	GCA_015472975.1
	Rhinolophidae	Rhinolophus ferrumequinum	GCA_004115265.3
		Rhinolophus sinicus	GCA_001888835.1
	Hipposideridae	Hipposideros armiger	GCA_001890085.1
		Hipposideros galeritus	GCA_004027415.1
		Hipposideros pendleburyi	GCA_021464545.1
	Craseonycteridae	Craseonycteris thonglongyai	GCA_004027555.1

Supplementary Table 7. List of genomes used in the OrthoFinder analysis. The accession numbers of those genomes, the phylogenetic group these species belong to, and the IDs used in this study.

Species	Family	Accession	Annotated ID
Artibeus intermedius	Phyllostomidae	GCA_038363225.1	Artibeus_intermedius
Artibeus lituratus	Phyllostomidae	GCA_038363175.3	Artibeus_lituratus
Artibeus jamaicensis	Phyllostomidae	GCA_021234435.1	Artibeus_jamaicensis_ref
Cnephaeus nilssonii	Vespertilionidae	GCA_951640355.1	Cnephaeus_nilssonii
Desmodus rotundus	Phyllostomidae	GCA_022682495.2	Desmodus_rotundus
Eptesicus fuscus	Vespertilionidae	GCA_027574615.1	Eptesicus_fuscus
Glossophaga mutica	Phyllostomidae	GCA_039655065.1	Glossophaga_mutica
Hipposideros armiger	Hipposideridae	GCA_001890085.1	Hipposideros_armiger
Hipposideros larvatus	Hipposideridae	GCA_031876335.1	Hipposideros_larvatus
Miniopterus natalensis	Vespertilionidae	GCA_001595765.1	Miniopterus_natalensis
Miniopterus schreibersii	Vespertilionidae	GCA_964146895.2	Miniopterus_schreibersii
Molossus molossus	Molossidae	GCA_014108415.1	Molossus_molossus
Molossus nigricans	Molossidae	GCA_039880945.1	Molossus_nigricans
Myotis daubentonii	Vespertilionidae	GCA_963259705.1	Myotis_daubentonii
Myotis myotis	Vespertilionidae	GCA_014108235.1	Myotis_myotis
Phyllostomus discolor	Phyllostomidae	GCA_004126475.3	Phyllostomus_discolor
Pipistrellus kuhlii	Vespertilionidae	GCA_014108245.1	Pipistrellus_kuhlii
Plecotus auritus	Plecotus	GCA_963455305.1	Plecotus_auritus
Pteronotus mesoamericanus	Mormoopidae	GCA_021234165.1	Pteronotus_mesoamericanus
Pteropus alecto	Pteropodidae	GCA_000325575.1	Pteropus_alecto
Pteropus vampyrus	Pteropodidae	GCA_000151845.2	Pteropus_vampyrus
Rhinolophus ferrumequinum	Rhinolophidae	GCA_004115265.3	Rhinolophus_ferrumequinum
Rhynchonycteris naso	Emballonuridae	GCA_037038545.1	Rhynchonycteris_naso
Rousettus aegyptiacus	Pteropodidae	GCA_014176215.1	Rousettus_aegyptiacus
Saccopteryx leptura	Emballonuridae	GCA_036850995.1	Saccopteryx_leptura
Sturnira hondurensis	Phyllostomidae	GCA_014824575.2	Sturnira_hondurensis

Supplementary Table 9. List of primers and probes for the target genes to evaluate host gene expression changes in *Artibeus jamaicensis* cells

Primer/probe name	Sequence
ISG15 F	CAGAAGGTGGCTGAGCTGAA
ISG15 P	FAM-TGGCTGAGTTTCCAGGGGAGGCCC
ISG15 R	CTTGTATTCCTTCAGCTGCGC
Ifit1 F	AGAGCTTGAAGCAGGCTGAA
Ifit1 P	FAM-ACATGCTGGCCAGTCGGAGGTGAG
Ifit1 R	CTTCTAGTCTGCCCATGCGG
Mx1 F	GTTCTTCATGCTCCGGTCGT
Mx1 P	FAM-GCCAGAAGCTGAGCAATGCCATGTTGC
Mx1 R	TTCCTCTTGTCGCTGGTGTC
OAS1 F	CGTGGTGCAGAGTCACAGAT
OAS1 P	FAM-CGCGTGATACGGAAACCTCGCTCGC
OAS1 R	GGGCAGGACATCAAACTCCA
Ddx58 F	GGAAGAACCCGAATGCCAGA
Ddx58 P	FAM-CAGCCCACTGAAGCCCAGAAATTAC
Ddx58 R	TTGATAGCAGGAGCAGCGAG
HPRT F	AGATGGTGAAGGTCGCAAG
HPRT P	FAM-ACTTTGTTGGATTTGAAATTCCAGACAAGTTTG
HPRT R	CCTGAAGTATTCATTATAGTCAAGGG

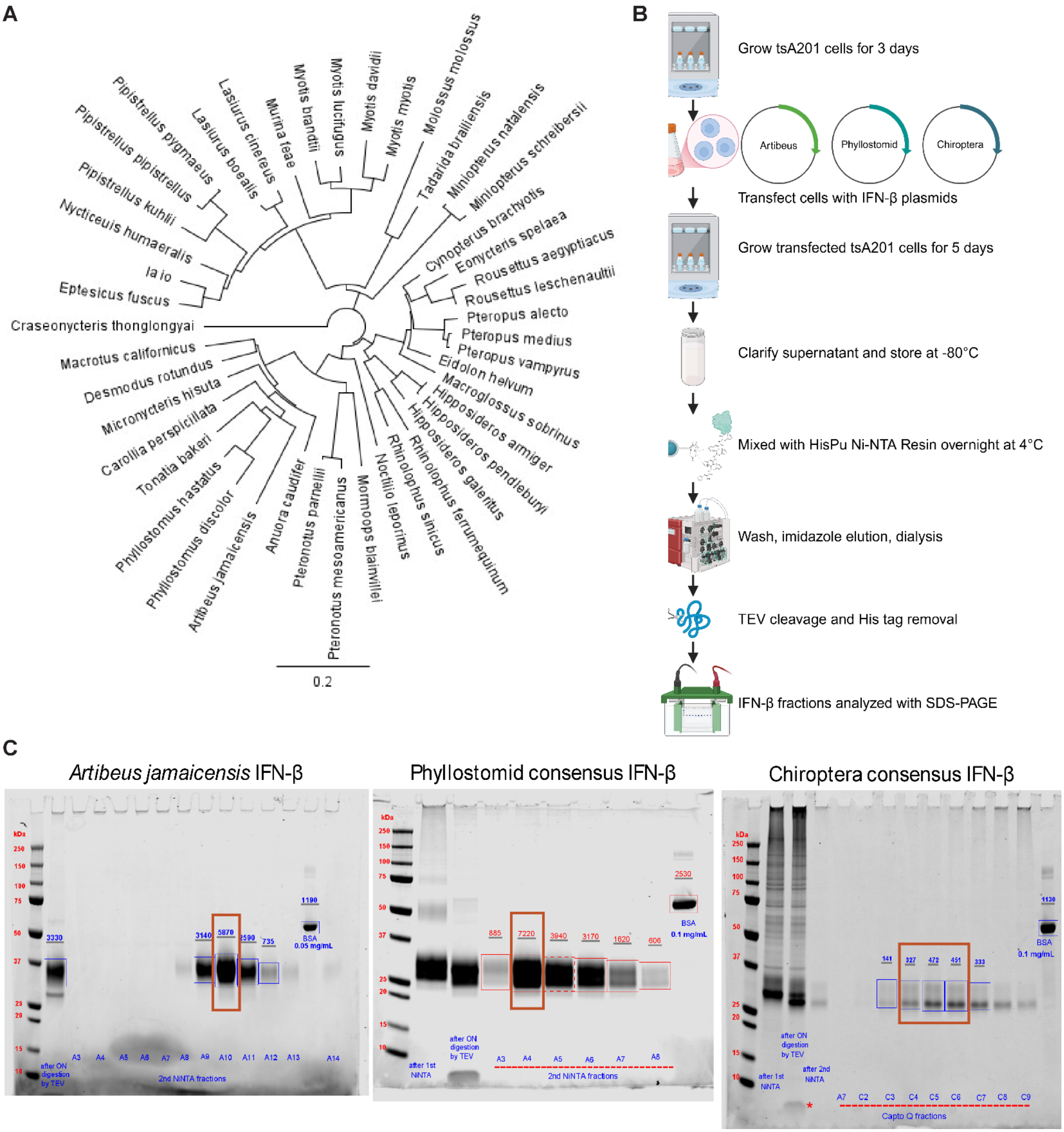

A
Molossus molossus
Tadarida brailiensis
Miniopterus natalensis
Miniopterus schreibersii
Cynopterus brachyotis
Eonycteris spelaea
Rousettus aegyptiacus
Rousettus leschenaultii
Pteropus alecto
Pteropus medius
Pteropus vampyrus
Eidolon helvum
Macroglossus sobrinus
Hipposideros armiger
Hipposideros pendleburyi
Hipposideros galeritus
Rhinolophus ferrumequinum
Rhinolophus sinicus
Noctilio leporinus
Mormoops blainvillei
Pteronotus mesoamericanus
Pteronotus parnellii
Anuora caudifer
Artibeus jamaicensis
Phyllostomus discolor
Phyllostomus hastatus
Tonatia bakeri
Carollia perspicillata
Micronycteris hisuta
Desmodus rotundus
Macrotus californicus
Craseonycteris thonglongyai
Eptesicus fuscus
Ia io
Nycticeuis humaeralis
Pipistrellus kuhlii
Pipistrellus pipistrellus
Pipistrellus pygmaeus
Lasiurus boealis
Lasiurus cinereus
Murina feae
Myotis brandtii
Myotis lucifugus
Myotis davidii
Myotis myotis
0.2
B
Grow tsA201 cells for 3 days
Artibeus
Phyllostomid
Chiroptera
Transfect cells with IFN-β plasmids
Grow transfected tsA201 cells for 5 days
Clarify supernatant and store at -80°C
Mixed with HisPu Ni-NTA Resin overnight at 4°C
Wash, imidazole elution, dialysis
TEV cleavage and His tag removal
IFN-β fractions analyzed with SDS-PAGE
C
Artibeus jamaicensis IFN-β
kDa
250
150
100
75
50
37
25
20
15
10
3330
3140
5870
2590
735
1190
BSA
0.05 mg/mL
after ON digestion by TEV
A3
A4
A5
A6
A7
A8
A9
A10
A11
A12
A13
A14
2nd NiNTA fractions
Phyllostomid consensus IFN-β
kDa
250
150
100
75
50
37
25
20
15
10
885
7220
3940
3170
1620
606
2530
BSA
0.1 mg/mL
after 1st NiNTA
after ON digestion by TEV
A3
A4
A5
A6
A7
A8
2nd NiNTA fractions
Chiroptera consensus IFN-β
kDa
250
150
100
75
50
37
25
20
15
10
141
327
472
451
333
1130
BSA
0.1 mg/mL
after ON digestion by TEV
after 1st NiNTA
after 2nd NiNTA
A7
C2
C3
C4
C5
C6
C7
C8
C9
Capto Q fractions

Supplementary Fig 1. Generation of recombinant bat IFN-βs

(A) Phylogenetic tree representing the species used to generate the Chiroptera consensus IFN-β, the full list of species and accession numbers for the genomes used is presented in Sup. Table 1. (B) Schematic of the protein synthesis and purification pipeline used to generate the recombinant bat IFN-βs. (C) Protein gel showing the purified IFN-β fractions that were purified. The dark red box indicates the fraction or fractions that were used for each of the recombinant bat IFN-βs.

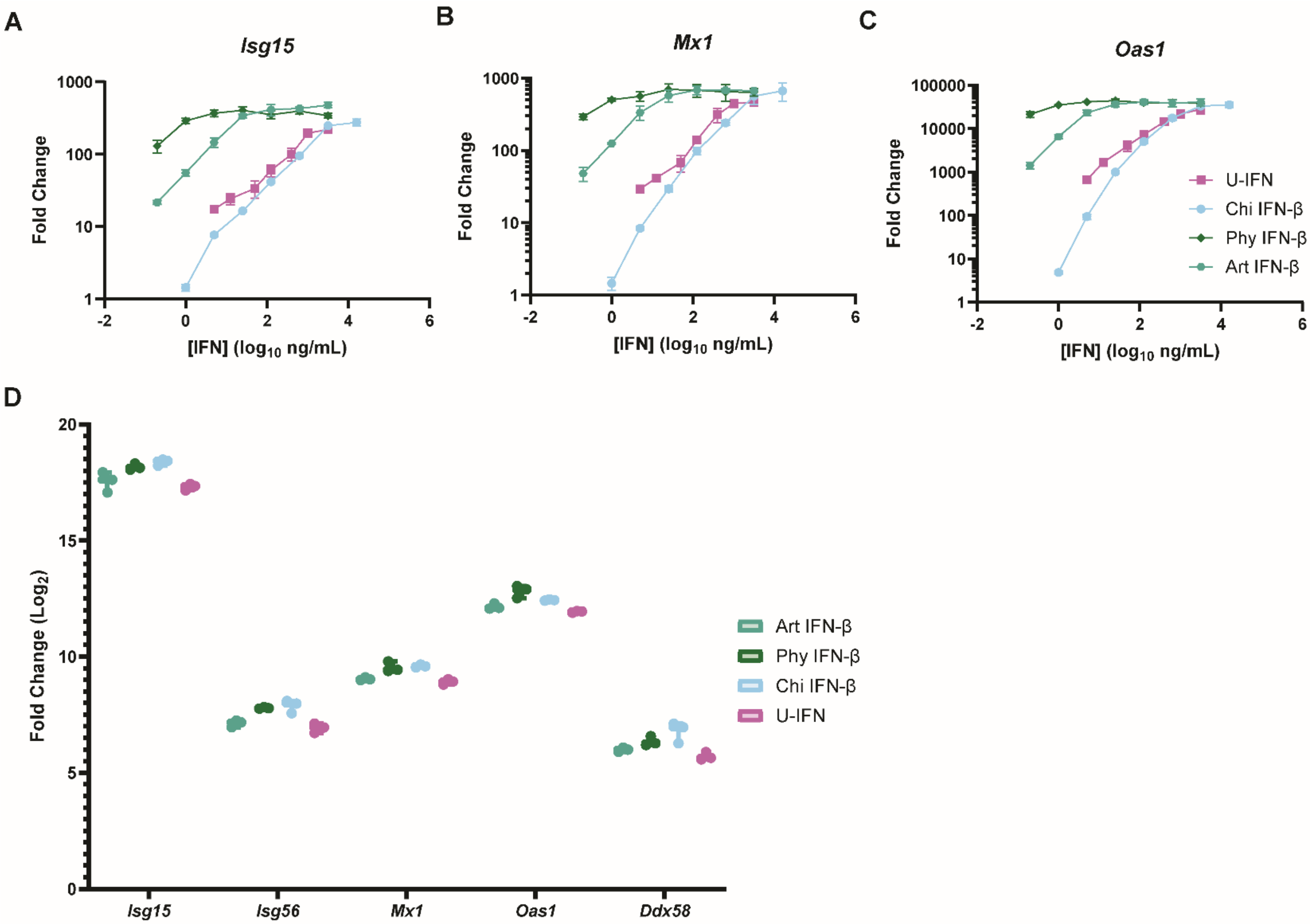

Supplementary Fig 2. Determination of functionally equivalent doses to compare interferon stimulated gene repertoires

(A-C) Primary Jamaican fruit bat cells cells were stimulated with serial dilutions of *Artibeus jamaicensis* (Art), Phyllostomid (Phy), or Chiroptera (Chi) interferon-β (IFN- β) or universal type I IFN (U-IFN) for 16 hours then their RNA was collected and evaluated for fold changes of IFN-stimulated genes (ISG), *Isg15* (A), *Mx1* (B), and *Oas1* (C). (D) AjKi2 cells were stimulated with Artibeus- (n=3), Phyllostomid- (n=3), or Chiroptera- (n=3), IFN-β or universal IFN (n-3) for 16 hours then expression of ISGs was assessed and compared to mock stimulated cells (n=3) using RT-qPCR prior to sending the RNA samples for transcriptomics.

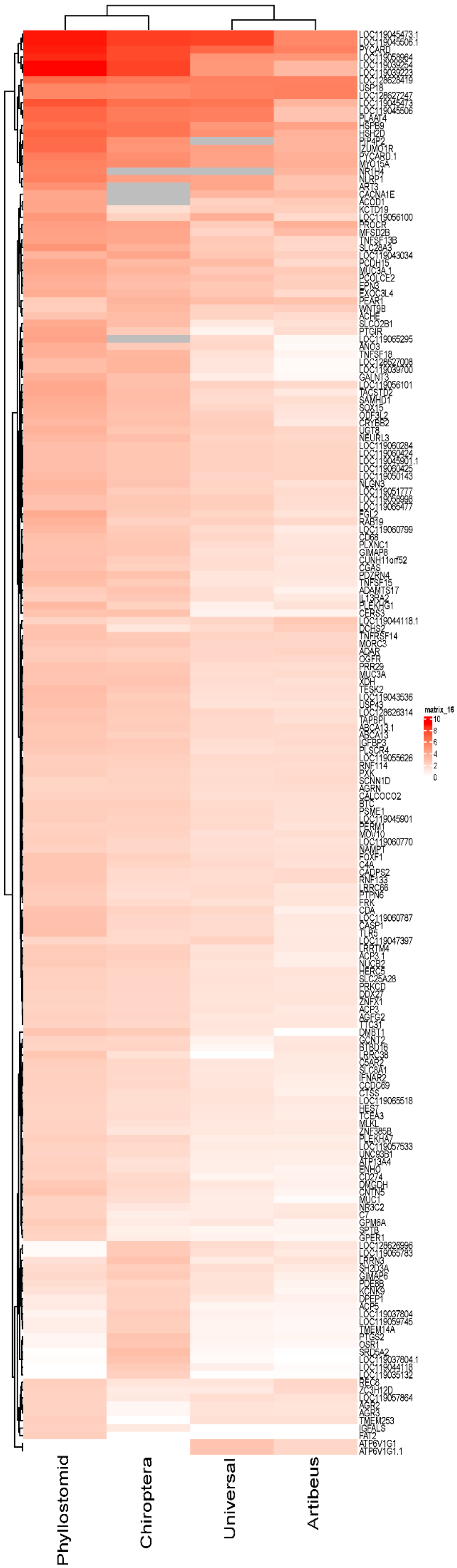
Phyllostomid
Chiroptera
Universal
Artibeus
matrix_16
10
8
6
4
2
0

Supplementary Fig 3. Differentially induced genes share similar expression patterns across the IFN-I treatment groups

Primary AjKi2 cells were stimulated with Artibeus- (n=3), Phyllostomid- (n=3), or Chiroptera- (n=3), IFN-β or universal IFN (n-3) for 16 hours then their transcriptomes were assessed and compared to mock stimulated cells (n-3). The genes (n= 202) upregulated by at least one, but not all, IFN-I types are presented as a heat map.

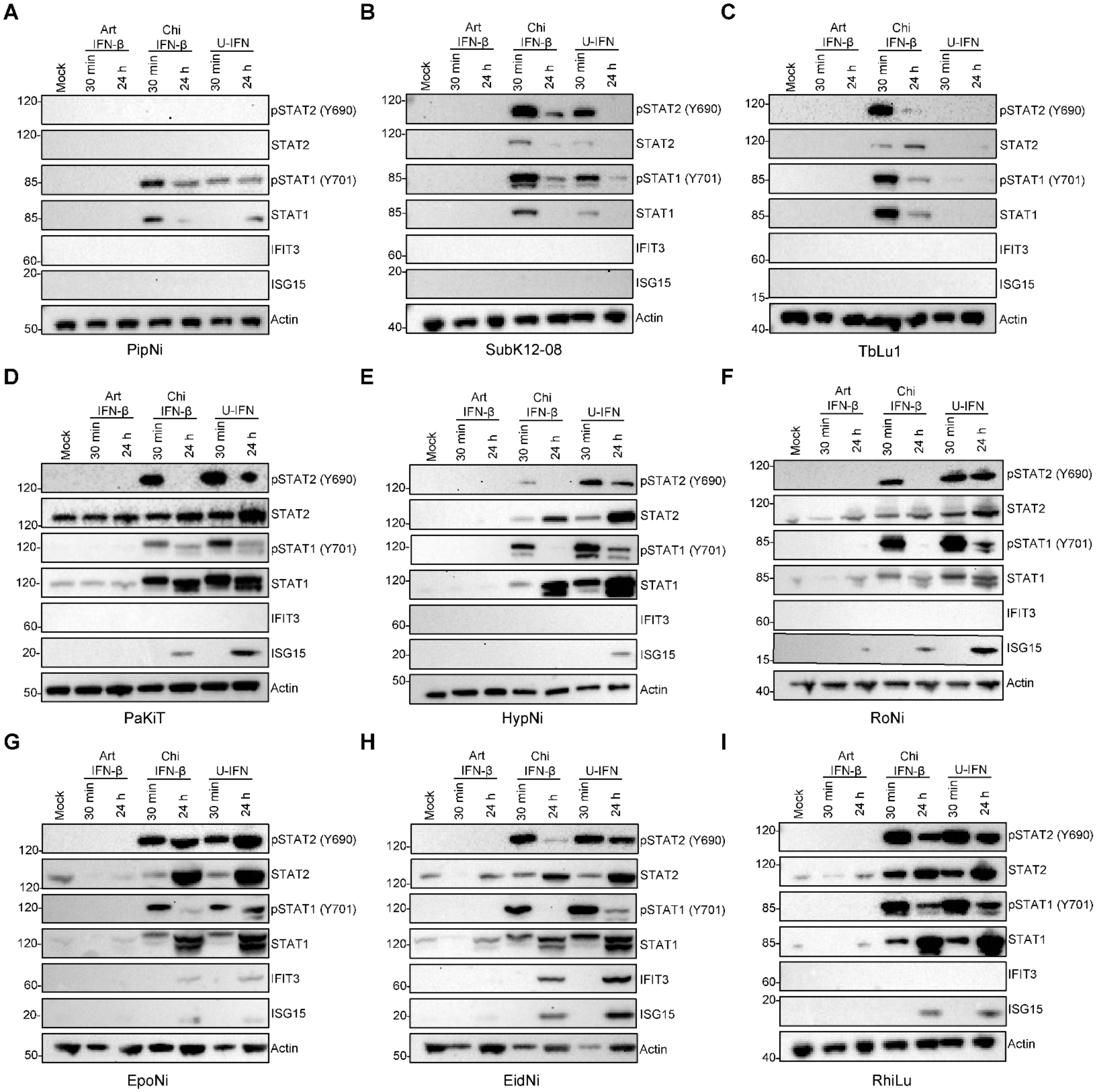
A
B
C
D
E
F
G
H
I
Art IFN-β
Chi IFN-β
U-IFN
Mock
30 min
24 h
pSTAT2 (Y690)
STAT2
pSTAT1 (Y701)
STAT1
IFIT3
ISG15
Actin
120
85
60
50
40
20
15
PipNi
SubK12-08
TbLu1
PaKiT
HypNi
RoNi
EpoNi
EidNi
RhiLu

Supplementary Fig 4. Chiroptera IFN-β induces IFN-I signaling in cell lines from diverse bat taxa

Immunoblots of PipNi (A), SubK12-08 (B), TbLu-1 (C), PaKiT (D), HypNi (E), RoNi (F), EpoNi (G), EidNi (H), and RhiLu (I) cells treated with *Artibeus jamaicensis* (Art), Chiroptera (Chi) IFN-β, or Univeral type I IFN (U-IFN) (500 ng/mL) for 30 minutes (min) or 24 hours (h). Blots were probed with phosphorylated (p) STAT1 and STAT2 to evaluate activation of the IFN-I receptor, and STAT1, STAT2, IFIT3, and ISG15 are prototypical IFN-stimulated genes. β-actin was also evaluated as a loading control. Blots shown are representative of at least three independent experiments and were used to calculate densitometry shown in Fig. 3 A-I.

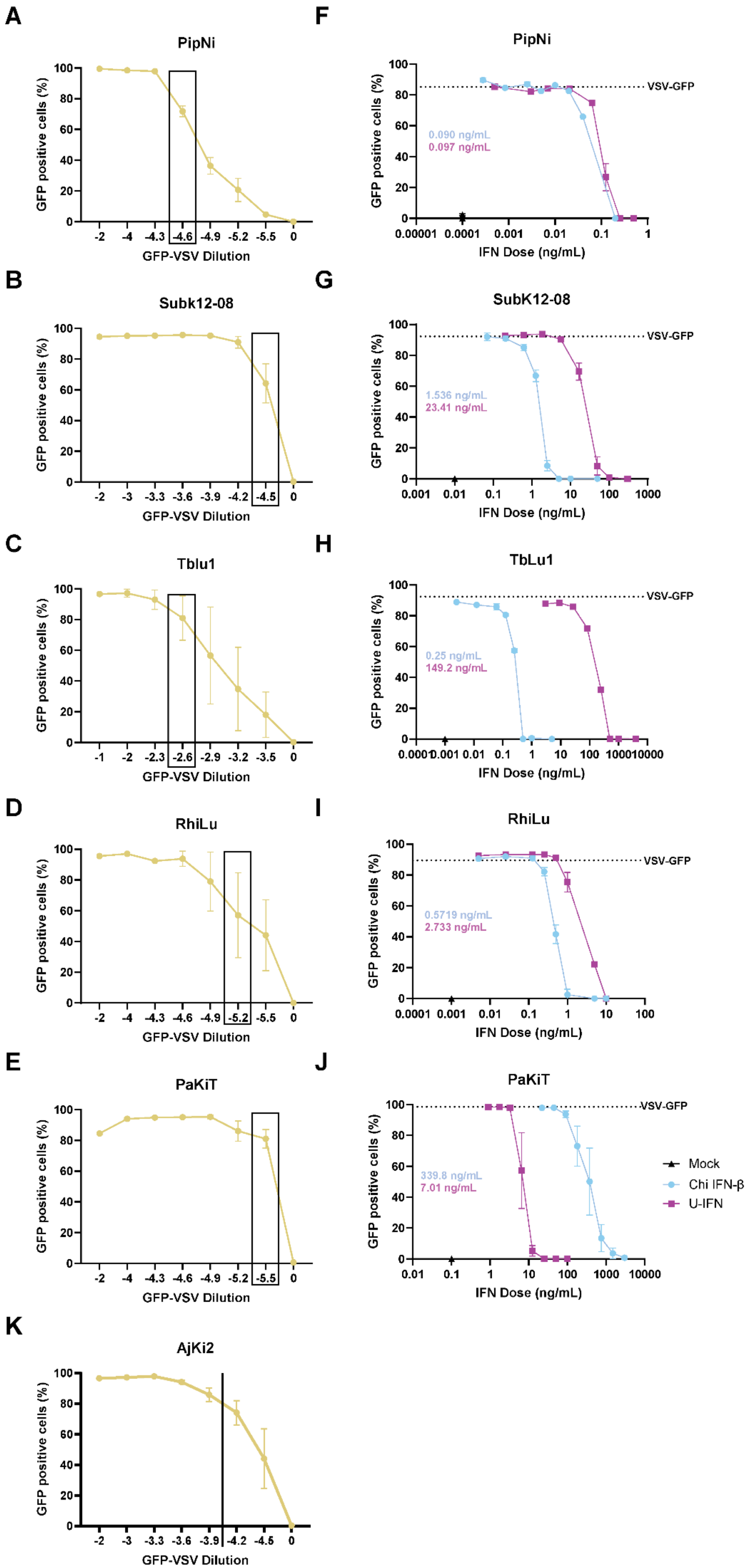

A
PipNi
GFP positive cells (%)
GFP-VSV Dilution
-2 -4 -4.3 -4.6 -4.9 -5.2 -5.5 0
B
Subk12-08
GFP positive cells (%)
GFP-VSV Dilution
-2 -3 -3.3 -3.6 -3.9 -4.2 -4.5 0
C
Tblu1
GFP positive cells (%)
GFP-VSV Dilution
-1 -2 -2.3 -2.6 -2.9 -3.2 -3.5 0
D
RhiLu
GFP positive cells (%)
GFP-VSV Dilution
-2 -4 -4.3 -4.6 -4.9 -5.2 -5.5 0
E
PaKiT
GFP positive cells (%)
GFP-VSV Dilution
-2 -4 -4.3 -4.6 -4.9 -5.2 -5.5 0
K
AjKi2
GFP positive cells (%)
GFP-VSV Dilution
-2 -3 -3.3 -3.6 -3.9 -4.2 -4.6 0
F
PipNi
VSV-GFP
0.090 ng/mL
0.097 ng/mL
GFP positive cells (%)
IFN Dose (ng/mL)
G
SubK12-08
VSV-GFP
1.536 ng/mL
23.41 ng/mL
GFP positive cells (%)
IFN Dose (ng/mL)
H
TbLu1
VSV-GFP
0.25 ng/mL
149.2 ng/mL
GFP positive cells (%)
IFN Dose (ng/mL)
I
RhiLu
VSV-GFP
0.5719 ng/mL
2.733 ng/mL
GFP positive cells (%)
IFN Dose (ng/mL)
J
PaKiT
VSV-GFP
339.8 ng/mL
7.01 ng/mL
GFP positive cells (%)
IFN Dose (ng/mL)
Mock
Chi IFN-β
U-IFN

Supplementary Fig 5. Chiroptera IFN-β induces an antiviral state in cell lines from diverse bat taxa

(A-E) VSV-GFP doses yielding approximately 70–90 percent (%) GFP-positive single cells for each cell line were selected: PipNi ($10^{-4.6}$) (A), SubK12-08 ($10^{-4.5}$) (B), TbLu-1 ($10^{-2.6}$) (C), RhiLu ($10^{-5.2}$) (D) cells, and 1 PaKiT cells ($10^{4.6}$) (E). (F-J) Flow cytometry was used to quantify the percentage of single cells positive for GFP following treatment with a dilution series of IFNs and a single dose of VSV-GFP. IC50 values were calculated for Chi IFN-β and U-IFN for each cell line. Presented data a representative of at least three independent experiments conducted in quadruplicate. These data were used to calculate the fold difference in IC50 presented in Fig. 3O.

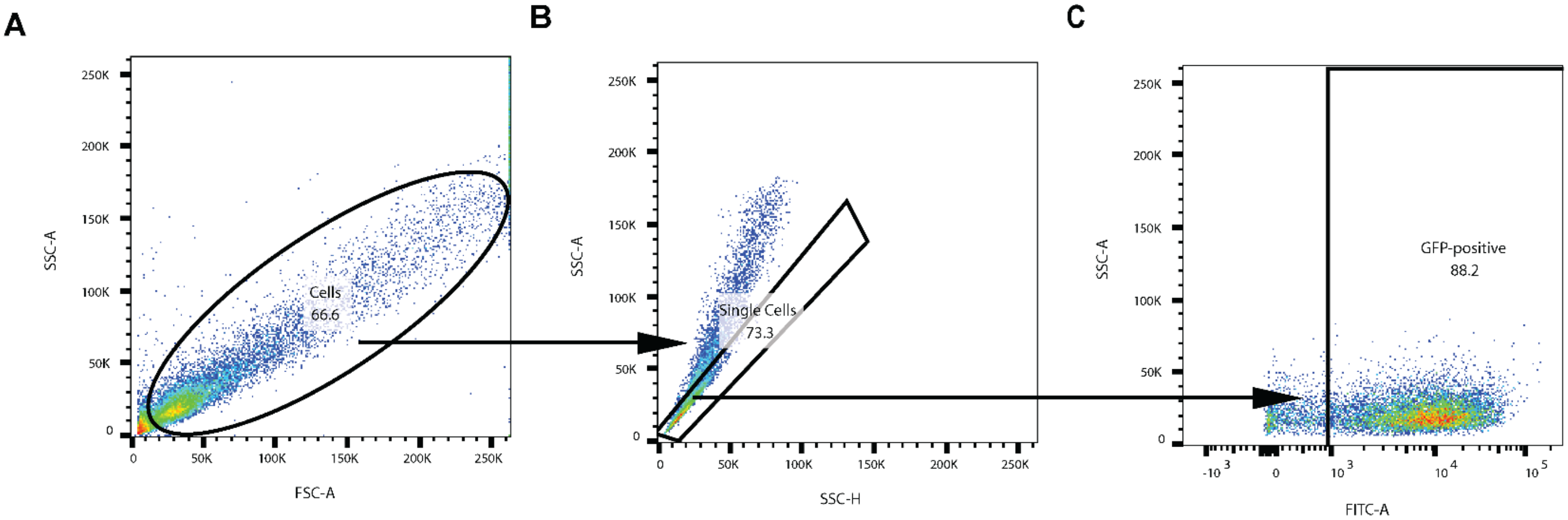

Supplementary Fig 6. Gating strategy to evaluate infection with VSV- or EBOV-GFP

Representative gating strategy for antiviral assays. (A) Cells were gated on SSC-A vs FSC-A to exclude debris and dead cells. (B) A SSC-A vs SSC-H gate was applied to limit analysis to single cells. (C) Singlets were then gated on FSC-A vs FITC-A to determine the percent of VSV-GFP-positive single cells. Strategy was applied to data presented in Fig 1G-H and Supp Fig 5A-J.
